# Comparison of hand-assisted laparoscopic surgery and conventional laparotomy for colorectal cancer: Interim results from a single institution

**DOI:** 10.3892/ol.2014.2182

**Published:** 2014-05-27

**Authors:** TAKAYUKI TAJIMA, MASAYA MUKAI, MASASHI YAMAZAKI, SHIGEO HIGAMI, SOUICHIROU YAMAMOTO, SAYURI HASEGAWA, EIJI NOMURA, SOTARO SADAHIRO, SEIEI YASUDA, HIROYASU MAKUUCHI

**Affiliations:** 1 Department of Surgery, Tokai University School of Medicine, Bohseidai, Isehara, Kanagawa 259-1193, Japan; 2 Department of Surgery, Tokai University Hachioji Hospital, Hachioji, Tokyo 192-0032, Japan

**Keywords:** colorectal cancer, HALS, CL, LACS, laparoscopic surgery

## Abstract

The present study aimed to compare the results of hand-assisted laparoscopic surgery (HALS) and conventional laparotomy (CL) at a single institution in Japan. Of the 212 patients with stage I/II/III colorectal cancer who received a curative resection, 98 patients underwent HALS and 114 patients underwent CL. The clinical background and post-operative management did not differ between the two groups. There were no significant differences in the 3-year relapse-free and 3-year overall survival rates between the HALS and CL groups for the patients in any stage. Blood loss during surgery was 250.1 and 135.5 ml (mean and median; the same hereafter) in stage I patients receiving HALS versus 608.2 and 315.5 ml in stage I CL patients (P=0.006), while it was 277.6 and 146 ml in stage II patients receiving HALS versus 548.6 and 347 ml in stage II CL patients (P=0.004). Post-operative hospital stay was recorded at 16.8 and 15 days in stage III patients receiving HALS versus 23.1 and 21 days in stage III CL patients (P=0.001). There were no significant differences in the operating time or complications between the two groups. These results indicate that the survival rate was comparable for HALS and CL, while HALS caused less surgical stress and achieved a better cosmetic outcome. The results of the final analysis of this cohort are awaited.

## Introduction

Less invasive laparoscopic surgery has become extremely popular in recent years, and is widely employed for tumor resection in patients with early stage I colorectal cancer, for radical curative resection in patients with more advanced colorectal cancer (stage II/III) and for palliative surgery in stage IV patients (1–6). As the deep pelvic floor, lower aspect of the bladder and the area posterior to the apex of the prostate are difficult to observe during conventional laparotomy (CL), manipulation has to be conducted almost blind. By contrast, a laparoscope provides a magnified perspective of the surgery that can be viewed on a monitor, resulting in a safer procedure (4).

Laparoscopy-assisted colorectal surgery (pure LACS) is the mainstream procedure in Japan; it requires 5 to 6 ports, including a camera port, together with a small incision of 35–45 mm for the anastomosis. With pure-LACS, surgical procedures that require 4 forceps are common, which means that at least 2 operators familiar with LACS are required. The long operating time can also be problematic due to the limited availability of anesthesiologists, operating theaters and theater staff. Expensive training in the technical procedures of LACS, and the cost of materials and instruments are other problems associated with pure-LACS at medium-sized hospitals of 400 to 500 beds (4–8). Compared with CL, pure-LACS has been reported to achieve fewer wound infections, a shorter hospital stay, comparable or better survival and a superior cosmetic outcome (7–10).

By contrast, surgeons in Europe and the USA generally perform hand-assisted laparoscopic surgery (HALS) or hybrid HALS (HH), which is combined with open procedures as necessary. HH has many advantages such as: i) Safe palpation and manipulation with the left hand similar to CL, so that large and heavy tumors can be resected en bloc and smoothly; ii) a shorter operating time compared with LACS; and iii) no need for extensive training to master the required skills (8,9,11–17). In Japan, HH has been used for the extended resection of two or more colorectal fields, for subtotal/total colectomy in patients with ulcerative colitis or Crohn’s disease and for simultaneous resection of multiple tumor foci, such as a lateral segmentectomy with splenectomy.

Surgery for colorectal cancer in Japan is performed by pure-LACS in 30–40% of patients, by CL in ~50% and by HALS or mini-laparotomy in the remaining patients (18). When HALS and pure-LACS are compared, the operating time is shorter and the conversion rate to CL is lower with HALS. HALS is considered to be a method that achieves results that fall between those of CL and pure-LACS (8,9,19–23). HALS became popular in Japan around the year 2000, at the time when pure-LACS was introduced, however, its use has declined markedly, as pure-LACS has become standardized in recent years. Single-center reports on HALS have been published overseas, but not in Japan (9,24). Accordingly, the purpose of the present study was to compare the results of HALS and CL at a single institution in Japan.

## Patients and methods

### Patients

A total of 850 patients underwent radical curative resection of primary colorectal cancer at the Tokai University Hachioji Hospital (Tokyo, Japan) between April 2002 and December 2012. HALS was actively employed for patients with colorectal cancer from July 2007 and was used in at least 350 patients. From the 850 patients, 114 patients (27 in stage I, 44 in stage II, and 43 in stage III) who underwent conventional radical resection by CL prior to July 2007 when HALS was introduced, were selected as stage-matched historical controls, while the HALS group was comprised of 98 patients who underwent HALS after July 2007 (41 in stage I, 29 in stage II, and 28 in stage III) (Table I). The two groups received the same post-operative adjuvant chemotherapy and follow-up regimen. The patients in the groups had a performance status of 0–2, no serious cardiac or pulmonary disease, no lateral lymph node metastasis of the pelvic cavity or multiple organ involvement documented prior to surgery and no bulky tumor in the pelvic cavity (4,25,26). The study was approved by the the institutional review board of the Tokai University Hachioji Hospital (Tokyo, Japan). All patients provided written informed consent.

### Treatment

Surgery was performed via a typical midline laparotomy incision at least 30 cm in length in the CL group. For HALS, a small incision of 45 to 55 mm was made in the midline above the umbilicus (colon) or the umbilical region (rectosigmoid lesion and rectum) prior to establishing 2 ports (colon, 5/5 mm) or 3 ports (rectosigmoid and rectum, 5/12/5 mm) (Table I) (4,25,26). A standardized D2 or D3 resection was performed for all patients in each group, with at least 12 lymph nodes being harvested according to the General Rules for Clinical Studies on Cancer (27–29). Stage I patients received no post-operative adjuvant chemotherapy, stage II patients received oral anticancer therapy (400 mg/m^2^ tegafur/uracil and 3 g polysaccharide K on five consecutive days per week for at least six months) and stage III patients received modified 5-fluorouracil (5FU)/leucovorin (LV) or modified FOLFIRI (5FU/LV+irinotecan; 60 mg/m^2^ irinotecan twice a month, and 350 mg/m^2^ 5-FU and 150 mg/m^2^ LV on five consective days per month) for at least six months (30–35).

### Survival

Metastasis/recurrence was detected by performing ultrasound and/or CT scanning 3 to 4 times a year, and patients in whom metastasis/recurrence was detected by imaging modalities were judged to have metastasis/recurrence (30–35). The 3-year relapse-free survival (3Y-RFS) and 3-year overall survival (3Y-OS) rates were calculated separately for stage I, II, and III patients in each group. Mean and median values were calculated for blood loss, operating time and post-operative hospital stay, and the re-operation rate with conversion rate to CL was also determined in the HALS group. Intergroup comparisons were also performed for post-operative complications, including wound infection, ileus and anastomotic leakage.

### Statistical analysis

3Y-RFS and 3Y-OS were calculated by the Kaplan-Meier method, while the log-rank test and hazard ratios [95% confidence interval (CI)] were used for comparisons between the two groups. The χ^2^ test and the Mann-Whitney U test were used for the other analyses. SPSS statistics 17.0 software (IBM SPSS, Armonk, NY, USA) was employed and P<0.05 was considered to indicate a statistically significant difference.

## Results

### Stage and survival rates

**Figure 1:**
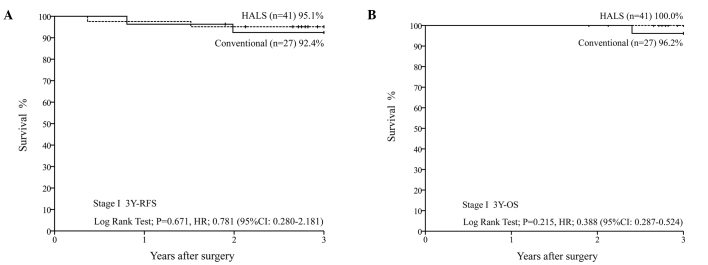
(A) 3-year relapse-free survival (3Y-RFS) rate and (B) 3-year overall (3Y-OS) rate of stage I patients who underwent HALS or CL, as estimated by the Kaplan-Meier method and log-rank test. The HR (95% CI) was also calculated. HALS, hand-assisted laparoscopic surgery; CL, conventional laparotomy; HR, hazard ratio; CI, confidence interval.

**Figure 2:**
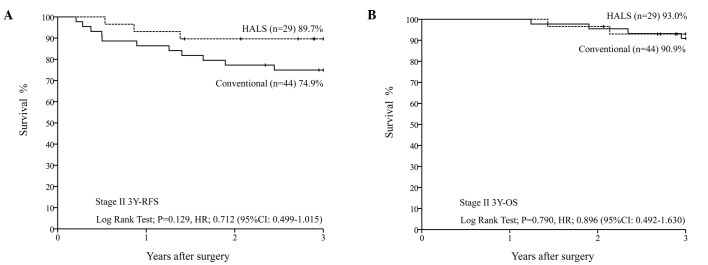
(A) 3-year relapse-free survival (3Y-RFS) rate and (B) 3-year overall survival (3Y-OS) rate of stage II patients who underwent HALS or CL, as estimated by the Kaplan-Meier method and the log-rank test. The HR (95% CI) was also calculated. HALS, hand-assisted laparoscopic surgery; CL, conventional laparotomy; HR, hazard ratio; CI, confidence interval.

**Figure 3:**
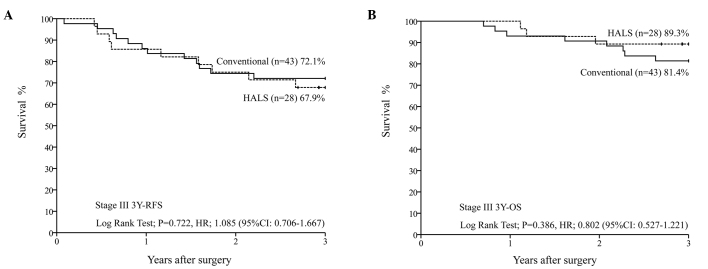
(A) 3-year relapse-free survival (3Y-RFS) rate and (B) 3-year overall survival (3Y-OS) rate of stage III patients who underwent HALS or CL, as estimated by the Kaplan-Meier method and the log-rank test. The HR (95% CI) was also calculated. HALS, hand-assisted laparoscopic surgery; CL, conventional laparotomy; HR, hazard ratio; CI, confidence interval.

A total of 68 patients exhibited stage I disease. The 3Y-RFS rate was 95.1% for the 41 HALS patients and 92.4% for the 27 CL patients [P=0.671; hazard ratio (HR), 0.781 95% CI, 0.280–2.181; Fig. 1A], whereas the 3Y-OS rate was 100.0% for the HALS patients versus 96.2% for the CL patients (P=0.215; HR, 0.388; 95% CI, 0.287–0.524; Fig. 1B). A total of 73 patients exhibited stage II disease. The 3Y-RFS rate was 89.7% for the 29 HALS patients and 74.9% for the 44 CL patients (P=0.129; HR, 0.712; 95% CI, 0.499–1.015; Fig. 2A), whereas the 3Y-OS rate was 93.0% for the HALS patients versus 90.9% for the CL patients (P=0.790; HR, 0.896; 95% CI, 0.492–1.630; Fig. 2B). There were a total of 71 patients with stage III disease. The 3Y-RFS rate was 67.9% for the 28 HALS patients and 72.1% for the 43 CL patients (P=0.722; HR, 1.085; 95% CI, 0.706–1.667; Fig. 3A), while the 3Y-OS rate was 89.3% for the HALS patients versus 81.4% for the CL patients (P=0.386; HR, 0.802; 95% CI, 0.527–1.221; Fig. 3B).

### Surgical results and hospital stay

Data for blood loss, operating time and hospital stay are presented as mean/median (range).

#### Stage I patients

The level of intraoperative blood loss was 250.1/135.5 (4–2,400 ml) in the 41 stage I patients who underwent HALS, whereas it was 608.2/315.5 (32–4,293 ml) in the 27 stage I patients who underwent CL (P=0.006) (Table II). The operating time was 3 h 5 min/3 h 10 min (1 h 10 min–5 h 57 min) for the HALS patients, while it was 3 h 25 min/3 h 18 min (1 h 40 min–5 h 23 min) for the CL patients (P=0.214) (Table II). The duration of post-operative hospital stay was 22.9/15 (9–177 days) for the HALS patients versus 23.3/17 (10–97 days) for the CL patients (P=0.260) (Table II).

#### Stage II patients

The level of intraoperative blood loss was recorded as 277.6/146 (9–1,354 ml) for the 29 stage II HALS patients versus 548.6/347 (37–1,913 ml) for the 44 stage II CL patients (P=0.004) (Table II). The operating time was 3 h 14 min/2 h 53 min (1 h 45 min–6 h 21 min) for the HALS patients, while it was 3 h 20 min/3 h 14 min (1 h 58 min–5 h 11 min) for the CL patients (P=0.282) (Table II). The duration of post-operative hospital stay was 19.8/16 (10–44 days) for the HALS patients and 20.7/17 (9–50 days) for the CL patients (P=0.381) (Table II).

#### Stage III patients

The level of intraoperative blood loss was recorded as 213.1/190 (42–483 ml) for the 28 HALS patients in stage III versus 417.3/229 (19–1,951 ml) for the 43 CL patients in Stage III (P=0.107) (Table II). The operating time was 3 h 13 min/3 h 07 min (1 h 53 min–5 h 25 min) for the HALS patients, whereas it was 3 h 07 min/3 h 06 min (1 h 39 min–5 h 43 min) for the CL patients (P=0.742) (Table II). The duration of post-operative hospital stay was 16.8/15 (9–47 days) for the HALS patients versus 23.1/21 (11–53 days) for the CL patients (P=0.001) (Table II).

### Complications

The post-operative complications in the HALS group (n=98) were wound infection in 11 patients (11.2%), ileus in 5 patients (5.1%), anastomotic leakage in 4 patients (4.1%), urinary tract injury in 3 patients (3.1%) and re-operation in 3 patients (3.1%), while the conversion rate to CL was 5.1% (5 patients) (Table III). Post-operative complications in the CL group (n=114) were wound infection in 17 patients (14.9%), ileus in 2 patients (1.8%), anastomotic leakage in 7 patients (6.1%), urinary tract injury in 5 patients (4.4%) and re-operation in 3 patients (2.6%). There were no significant differences in these events between the two groups (Table III).

## Discussion

In Japan, colorectal lesions are treated by pure LACS in 30 to 40% of patients, while CL is used for ~50% and the rest are managed by HALS and mini-laparotomy (20). With the widespread use of pure-LACS in recent years, comparisons of CL and HALS have been reported in numerous studies (8,9,19–23). However, the clinical background of the subjects can be an issue in such studies. A CL group is often selected as a control for the comparison of surgical procedures at a single site, but it is difficult to eliminate clinical bias in the selection of patients for pure-LACS and HALS. In other words, low-risk patients with a good general condition who can tolerate an oblique head-down position and early-stage patients are likely to be selected for pure-LACS, and it is difficult to standardize secondary management, including post-operative adjuvant chemotherapy or radiotherapy, and the treatment provided following recurrence and metastasis (4–10). When the National Clinical Database (http://www.ncd.or.jp/ established by the Japan Surgical Society) and the academic society guideline are used as a control, however, the study becomes a comparison with the national standards stratified by stage and is not suitable for comparison of different surgical procedures. Therefore, the present study used stage-matched historical controls from prior to the introduction of HALS, and the post-operative adjuvant chemotherapy was also standardized. All HALS and CL cases were performed by Mukai *et al* (4,26), therefore the therapeutic regimen for stage I/II/III rectal cancer cases may be standardized. The regimen for stage II/III was standardized, and at least 6 months of treatment was completed by >80% of patients in the two groups (data not shown). In addition, <20% of patients were censored for a lack of data/dropout, in agreement with the guideline for treatment of colorectal cancer in Japan. The database used in the present study was HALS (total, 11.9%) vs. CL (total, 1.8%) (P=0.001, data not shown), and the CL database consisted of control group subjects who were followed for nearly three years. The results of the final analysis will be available after another two years of HALS.

Compared with CL, pure-LACS has been shown to result in a longer operating time and much higher medical costs, although the hospital stay is shortened and the total use of analgesics is reduced (7–10). Problems such as the limited availability of physicians skilled in pure-LACS, the requirement for more training and the shortage of anesthesiologists, operating theaters and staff due to longer operations have been highlighted. These issues are problematic for medium-sized hospitals with 400–500 beds. Compared with HALS, a slow learning curve for mastering the technique, a longer operating time and a difference in the conversion rate to CL have been highlighted as problems with pure-LACS (8,9,19–23). The conversion rate has been reported to be far lower with HALS than pure-LACS, and it was only 5.1% (5/98 patients) in the present study (2,4,7). The five patients who underwent conversion consisted of two patients in stage I and three patients in stage II. As none of these patients were in stage III, it appears that the pre-operative diagnosis and indications for surgery were appropriate for stage III patients, although it should be kept in mind that radical curative resection may not be possible for N0 patients in stages I and II if CL is not performed. Compared with CL, less blood loss and a shorter hospital stay have been reported as advantages of HALS. In the present study, a significant difference was observed for the level of blood loss in the patients with stage I or II disease, as well as for the duration of the hospital stay in the patients with stage III disease. Such results could be anticipated, as bleeding obscures the operative field with laparoscopic surgery. Although the present study was stage-matched, stage III patients with resections of tumors involving multiple organs accounted for 18.6% (8/43 patients) of the CL group versus 3.6% (1/28 patients) of the HALS group (P=0.063; data not shown). It is possible that patients who were only suitable for CL were included in the CL group, and their hospital stay may have been prolonged due to more difficult surgery, although a significant difference was not observed. No significant difference in complications was found between the CL and HALS groups, but a detailed comparison by grade should be conducted in the future to assess the complications associated with surgical invasion.

In stage I/II patients, the volume of blood loss with HALS was lower than that with CL. This indicated that HALS was conducted safely based on the strict indications. HALS is a safe technique that allows conventional left hand manipulation in addition to palpation, and is a sensible compromise between pure-LACS and CL. HALS has an easy and low-cost method that can be an excellent option for use in medium to small hospitals throughout Japan, particularly in the current medical environment where the number of surgeons and anesthesiologists is decreasing.

## Figures and Tables

**Table I:** Comparison of stage I/II/III patients (n=212) who underwent HALS (n=98) or CL (n=114).

Surgical methods	HALS, % (n)	CL, % (n)	P-value^a^
Right hemicolectomy	26.5 (26)	24.6 (28)	0.743
Transverse colectomy	2.0 (2)	7.0 (8)	0.088
Left hemicolectomy	8.2 (8)	7.0 (8)	0.753
Sigmoidectomy			
Anterior resection	27.6 (27)	33.3 (38)	0.363
Lower anterior resection	30.6 (30)	21.1 (24)	0.111
Miles’ operation	5.1 (5)	7.0 (8)	0.562

a χ^2^ test.

HALS, hand-assisted laparoscopic surgery; CL, conventional laparotomy.

**Table II:** Surgical results and hospital stay of patients of differing stages who underwent HALS or CL.

A, Stage I patients (n=68)			

Surgical results and hospital stay	HALS (n=41)	CL (n=27)	P-value^a^
Blood loss, ml			
Mean	250.1	608.2	0.006
Median (range)	135.5 (4–2400)	315.5 (32–4293)	
Operating time			
Mean	3 h 05 min	3 h 25 min	0.214
Median (range)	3 h 10 min (1 h 10 min–5 h 57 min)	3 h 18 min (1 h 40 min–5 h 23 min)	
Post-operative hospital stay, days			
Mean	22.9	23.3	0.260
Median (range)	15 (9–177)	17 (10–97)	

B, Stage II patients (n=73)			

Surgical results and hospital stay	HALS (n=29)	CL (n=44)	P-value^a^

Blood loss, ml			
Mean	277.6	548.6	0.004
Median (range)	146 (9–1354)	347 (37–1913)	
Operating time			
Mean	3 h 14 min	3 h 20 min	0.282
Median (range)	2 h 53 min (1 h 45 min–6 h 21 min)	3 h 14 min (1 h 58 min–5 h 11 min)	
Post-operative hospital stay, days			
Mean	19.8	20.7	0.381
Median (range)	16 (10–44)	17 (9–50)	

C, Stage III patients (n=71)			

Surgical results and hospital stay	HALS (n=28)	CL (n=43)	P-value^a^

Blood loss, ml			
Mean	213.1	417.3	0.107
Median (range)	190 (42–483)	229 (19–1951)	
Operating time			
Mean	3 h 13 min	3 h 07 min	0.742
Median (range)	3 h 07 min (1 h 53 min–5 h 25 min)	3 h 06 min (1 h 39 min–5 h 43 min)	
Post-operative hospital stay, days			
Mean	16.8	23.1	0.001
Median (range)	15 (9–47)	21 (11–53)	

a Mann-Whitney U test;

HALS, hand-assisted laparoscopic surgery; CL, conventional laparotomy.

**Table III:** Post-operative complications in stage I/II/III patients (n=212) who underwent HALS (n=98) or CL (n=114).

Complications	HALS,% (n)	CL,% (n)	P-value^a^
Wound infection	11.2 (11)	14.9 (17)	0.429
Ileus	5.1 (5)	1.8 (2)	0.174
Leakage	4.1 (4)	6.1 (7)	0.500
Urinary tract injury	3.1 (3)	4.4 (5)	0.614
Re-operation	3.1 (3)	2.6 (3)	0.851
Others	6.1 (6)	5.3 (6)	0.787
Conversion to CL	5.1 (5)		
Stage I	2.0 (2)		
Stage II	3.1 (3)		
Stage III	0.0 (0)		

a χ^2^ test.

HALS, hand-assisted laparoscopic surgery; CL, conventional laparotomy.

## Abbreviations

HALS: hand-assisted laparoscopic surgery
CRC: colorectal cancer
CL: conventional laparotomy
LACS: laparoscopy-assisted colorectal surgery
